# Transcutaneous Auricular Vagus Nerve Stimulation for Postpartum Contraction Pain During Elective Cesarean Delivery: A Randomized Clinical Trial

**DOI:** 10.1001/jamanetworkopen.2025.29127

**Published:** 2025-08-29

**Authors:** Xingyu Xiong, Mingshu Tao, Wenxin Zhao, Wen Tan, Yanyu Jiang, Zhengxiu Sun, Yuanyuan Wang, Yongao Lin, Chunyan Li, Jie Yang, Yuan Han, Hongxing Zhang, Song Zhang, He Liu, Jun-Li Cao

**Affiliations:** 1 Department of Anesthesiology, the Affiliated Hospital of Xuzhou Medical University, Xuzhou, China; 2 National Medical Products Administration Key Laboratory for Research and Evaluation of Narcotic and Psychotropic Drugs, Xuzhou Medical University, Xuzhou, China; 3 Jiangsu Province Key Laboratory of Anesthesiology, Xuzhou Medical University, Xuzhou, China; 4 Jiangsu Key Laboratory of Applied Technology of Anesthesia and Analgesia, Xuzhou Medical University, Xuzhou, China; 5 Department of Anesthesiology, Eye & ENT Hospital of Fudan University, Shanghai, China; 6 Department of Anesthesiology, Renji Hospital and Shanghai Jiaotong University School of Medicine, Shanghai, China; 7 Department of Anesthesiology & Clinical Research Center for Anesthesia and Perioperative Medicine, Huzhou Central Hospital, Huzhou, China; 8 Department of Anesthesiology & Clinical Research Center for Anesthesia and Perioperative Medicine, The Fifth School of Clinical Medicine of Zhejiang Chinese Medical University, Huzhou, China; 9 Department of Anesthesiology & Clinical Research Center for Anesthesia and Perioperative Medicine, The Affiliated Huzhou Hospital, Zhejiang University School of Medicine, Huzhou, China; 10 Department of Anesthesiology & Clinical Research Center for Anesthesia and Perioperative Medicine, The Affiliated Central Hospital, Huzhou University School of Medicine, Huzhou, China; 11 Huzhou Key Laboratory of Basic Research and Clinical Translation for Neuromodulation, The Fifth School of Clinical Medicine of Zhejiang Chinese Medical University, Huzhou, China; 12 Ministry of Education Key Laboratory for Neuroinformation, University of Electronic Science and Technology of China, Chengdu, China

## Abstract

**Importance:**

Postpartum uterine contraction pain manifests as a severe, intermittent lower abdominal discomfort caused by uterine contraction during the early puerperium. Transcutaneous auricular vagus nerve stimulation (taVNS) has demonstrated promising effects in managing both acute and chronic pain conditions; however, there remains a paucity of studies regarding the use of taVNS for alleviating postpartum uterine contraction pain.

**Objective:**

To investigate the effect of taVNS in alleviating postpartum uterine contraction pain among women receiving combined spinal-epidural anesthesia for elective cesarean delivery.

**Design, Setting, and Participants:**

This randomized clinical trial was conducted from April 6 to August 31, 2024, at the Affiliated Hospital of Xuzhou Medical University, Xuzhou, China. Participants were aged 18 years or older and scheduled for elective cesarean delivery. Statistical analysis was performed in September 2024. All analyses were conducted according to the intention-to-treat principle.

**Interventions:**

Participants were randomly allocated 1:1 to either the active taVNS group or the sham taVNS group. Both groups received the stimulation for 30 minutes each time, once a day, on the day of surgery and postoperative days 1 and 2.

**Main Outcomes and Measures:**

The primary outcome was the incidence of moderate to severe postpartum uterine contraction pain on the third postoperative day, evaluated using a visual analogue scale (uterine contraction pain was classified as moderate to severe if the score was ≥4). Secondary outcomes included peak uterine contraction pain and incision pain, postpartum anxiety, postpartum depression, postpartum recovery quality, and sleep quality.

**Results:**

A total of 156 women were recruited and randomized to the active taVNS group (78 patients; mean [SD] age, 31.5 [4.3] years) or the sham taVNS group (78 patients; mean [SD] age, 31.3 [4.5] years). The incidence of moderate to severe uterine contraction pain in the active taVNS group was 5.1% (4 of 78) compared with 28.2% (22 of 78) in the sham taVNS group on the third postoperative day (relative risk, 0.18 [95% CI, 0.07-0.50]; *P* < .001) The active taVNS group had lower median scores on the third postoperative day for incisional pain (visual analogue score: 2.20 [IQR, 2.00-2.50] vs 3.00 [IQR, 2.60-3.33]), depression (EPDS: 3.00 [IQR, 2.00-4.00] vs 5.00 [IQR, 3.00-6.00]), and anxiety (PRAQ-R2: 13.50 [IQR, 12.00-15.00] vs 15.00 [IQR, 13.75-17.00]), as well as significantly higher scores for recovery quality on the third postoperative day (ObsQoR-11: 104 [IQR, 103-105] vs 99 [IQR, 96-101]) and sleep quality on the second postoperative day (LSEQ: 52.00 [IQR, 50.00-55.00] vs 47.50 [IQR, 43.00-52.00]) compared with the sham taVNS group.

**Conclusions and Relevance:**

In this randomized clinical trial, taVNS significantly reduced uterine contraction pain, incisional pain, depression, and anxiety, and improved recovery quality and sleep quality compared with sham taVNS. These findings suggest that taVNS represents a novel and safe strategy for alleviating postpartum uterine contraction pain among women undergoing cesarean delivery and for promoting postoperative recovery.

**Trial Registration:**

Chinese Clinical Trial Register Identifier: ChiCTR2400082716

## Key Points

**Question:**

What is the effect of transcutaneous auricular vagus nerve stimulation (taVNS) on postpartum uterine contraction pain among women undergoing elective cesarean delivery?

**Findings:**

This randomized clinical trial involving 156 women undergoing cesarean delivery found that 3 sessions of taVNS reduced the uterine contraction pain scores and incision pain scores during the 3 days postoperatively compared with sham stimulation.

**Meaning:**

These findings suggest that taVNS would be a safe and effective treatment for alleviating postpartum uterine contraction pain among women after cesarean delivery.

## Teaser

This randomized clinical trial compares the effect of transcutaneous auricular vagus nerve stimulation with that of sham stimulation in alleviating postpartum uterine contraction pain among women receiving combined spinal-epidural anesthesia for elective cesarean delivery.

## Introduction

Cesarean delivery is a critical, lifesaving surgical procedure used in situations such as prolonged labor, obstructed labor, fetal distress, or abnormal fetal positioning. Globally, the incidence of cesarean deliveries has shown a consistent upward trend.^1^ In addition to the common short-term and long-term risks associated with surgical procedures, including hemorrhage, infection, and prolonged recovery, women undergoing cesarean delivery also experience postpartum uterine contraction pain comparable with that experienced by women who deliver vaginally.^2,3^

Postpartum uterine contraction pain typically emerges within 1 to 2 days after delivery, characterized by intermittent and intense lower abdominal discomfort that may persist for several days.^4^ It is a visceral pain mediated by multiple mechanisms, with its pain signals transmitted through multiple spinal segments (involving both sympathetic and parasympathetic nerves).^5^ Local tissue ischemia induced by persistent uterine contractions gives rise to nociception.^6^ After cesarean delivery, the surgical incision of the uterus, along with the release of inflammatory mediators such as prostaglandins and histamine, significantly intensifies the pain associated with uterine contraction.^7^ The frequent and intense nature of uterine contraction is closely related to perioperative anxiety, depression, and sleep disturbances, which can adversely affect the quality of postpartum recovery and pose significant risks to the mother’s physical and psychological well-being.^8,9^ Moreover, the endogenous release of oxytocin during breastfeeding can exacerbate uterine contraction and pain, potentially delaying the initiation of breastfeeding and mother-infant, skin-to-skin contact, which may have implications for neonatal health.^10^ Therefore, it is imperative to address and mitigate the pain associated with uterine contraction after cesarean delivery.^11^

Currently, the most commonly used analgesic methods include epidural analgesia and intravenous administration of analgesics. Although these methods are effective, epidural analgesia during the postpartum period may restrict maternal mobility and increase the risk of infection, potentially resulting in complications such as pressure ulcers and permanent neurologic sequelae.^12,13^ Intravenous analgesics can cause adverse effects, including drowsiness, nausea, vomiting, pruritus, constipation, respiratory depression, acute opioid tolerance, and opioid-induced hyperalgesia.^14,15^ Moreover, these medications may affect the breastfed neonate, thereby imposing an additional psychological burden on the mother during lactation.^16^ Recently, nonpharmacologic interventions, such as acupuncture, acupressure, and neuromodulation techniques, have shown promising results in alleviating perioperative pain.^17,18,19^

Transcutaneous auricular vagus nerve stimulation (taVNS) is a noninvasive neuromodulation technique that delivers low-intensity electrical current through electrodes to stimulate the auricular branch of the vagus nerve. Transcutaneous auricular vagus nerve stimulation exerts analgesic effects by modulating the vagus nerve system and its projections to pain-specific brain nuclei^19,20^ and has also been shown to alleviate anxiety and depression.^21,22^

Transcutaneous auricular vagus nerve stimulation has been confirmed to activate the vagus nerve–nucleus tractus solitarius pathway and the cholinergic anti-inflammatory pathway, thereby regulating pain transmission and inhibiting inflammation.^23,24^ In addition, it helps reconstruct autonomic nerve balance,^25^ enhance vagal tone, and antagonize vasospasm caused by excessive sympathetic excitation.^26^ Therefore, we hypothesized that 3 sessions of taVNS may significantly reduce the incidence of moderate to severe uterine contraction pain among women receiving combined spinal-epidural anesthesia for elective cesarean delivery.

## Methods

### Study Design and Population

This randomized, double-blind, clinical trial was conducted at the departments of anesthesiology and obstetrics of the Affiliated Hospital of Xuzhou Medical University, Xuzhou, China, from April 6 to August 31, 2024. The study protocol was approved by the ethics committee of the Affiliated Hospital of Xuzhou Medical University and registered in the China Clinical Trial Registry on April 6, 2024. Written informed consent was obtained from all participants before their inclusion in the study. This study followed the Consolidated Standards of Reporting Trials (CONSORT) reporting guideline for randomized clinical trials. The trial protocol and statistical analysis plan are provided in Supplement 1.

### Participants

We recruited pregnant women aged 18 years or older, with a singleton pregnancy at 37 to 42 weeks of gestation, who were scheduled to undergo a cesarean delivery via a lower segment transverse incision under combined spinal-epidural anesthesia. Eligible participants were required to have an American Society of Anesthesiologists physical status classification of II or III. Exclusion criteria included refusal to sign the informed consent form, a history of neurologic or psychiatric disorders, cognitive impairment or communication difficulties, auricular dermatitis, substance abuse, or an inability to use patient-controlled intravenous analgesia. In addition, participants could withdraw from the study for reasons such as voluntary withdrawal, poor adherence, protocol deviations, or failure to complete follow-up.

### Randomization and Blinding

Participants were randomly allocated in a 1:1 ratio to either the active taVNS group or the sham taVNS group based on a computer-generated random number sequence. The allocation details were sealed in opaque envelopes and remained concealed until the researchers were ready to initiate the first intervention in the ward. All personnel involved in data collection, processing, and analysis, as well as other health care staff and participants, were blinded to the group assignments.

### Study Procedures and Interventions

On entering the operating room, a peripheral intravenous line was established, and standard monitoring of blood pressure, electrocardiography, and pulse oximetry were initiated. After skin disinfection, an experienced anesthesiologist performed combined spinal-epidural anesthesia after administering a local infiltration of 2% lidocaine. The lumbar puncture was conducted at the lumbar spine 2 (L2)–L3 or L3-L4 intervertebral spaces. For the intrathecal administration, 1.0 to 1.5 mL of 0.75% bupivacaine hydrochloride was intrathecally injected. Subsequently, the spinal needle was removed, and the epidural catheter was advanced 3 to 5 cm in the cephalad direction within the epidural space. If necessary, an additional 5 mL of 2% lidocaine was administered via the epidural catheter to provide supplemental analgesia. The maximum level of anesthesia was maintained at thoracic spine 4–thoracic spine 6, and then surgery was initiated. Throughout the procedure, oxygen was delivered via a nasal cannula at a flow rate of 2 L/min. After the delivery of the fetus, intravenous administrations of palonosetron, 0.075 mg, and nalbuphine hydrochloride, 10 mg, were performed. Vasopressor agents were administered as required to ensure hemodynamic stability during anesthesia. The epidural catheter was removed before the patient returned to the ward after the operation. Postoperative analgesia was administered via a patient-controlled intravenous analgesia (PCIA) pump. The pump was loaded with sufentanil, 1.5 µg/kg, nalbuphine, 30 mg, and palonosetron, 0.15 mg, which were diluted to 120 mL with normal saline. The patient-controlled intravenous analgesia pump had a loading dose of 2 mL, a basal infusion rate of 2 mL/h, and a self-controlled analgesic dose of 0.5 mL.

Participants were randomly assigned to receive either active taVNS or sham taVNS on the day of surgery and the first and second postoperative days after the administration of oxytocin in the ward. Electrical stimulation was applied through electrodes placed on the concha of the left ear. The apparatus used in this trial (tVNS501; Changzhou Ruishen’an Medical Devices Co Ltd) is shown in eFigure 1 in Supplement 2. In the active taVNS group, participants received taVNS (pulse width, 200 microseconds; frequency, 20 Hz) for 30 minutes, with the current increasing for 30 seconds until the participant reported a tingling sensation, then decreasing to a level slightly below this threshold for 30 minutes. In the sham taVNS group, the electrodes were positioned similarly, with the current increasing for 30 seconds until the participant reported a tingling sensation, then decreasing to a level slightly below this threshold, after which the device remained off for the following 30 minutes to simulate the sham state. Throughout the intervention, researchers closely monitored participants for any adverse effects and stopped the stimulation immediately if intolerable adverse effects were observed.

### Clinical Outcomes and Assessments

On the day before the operation (T0), researchers collected baseline data and prenatal information from women. The primary outcome was the incidence of moderate to severe uterine contraction pain on the third postoperative day (T7). Pain intensity was evaluated using a visual analogue scale (VAS). The VAS is a well-established tool for evaluating pain, consisting of a 100-mm line where one end represents no pain and the other end signifies the worst pain imaginable or the most extreme pain possible.^27^ Participants were asked to mark the point on the line that best corresponded to their perceived pain intensity. In this study, uterine contraction pain was classified as moderate to severe if the VAS score was 4 or higher.

Secondary outcomes included peak uterine contraction pain scores and incision pain scores during the 3 days postoperatively (assessed by the VAS; range, 0-10, where 0 indicates no pain and 10 indicates the worst possible pain). The pain scores were measured before the first intervention (T1), after the first intervention (T2), before the second intervention (T3), after the second intervention (T4), before the third intervention (T5), after the third intervention (T6), and on the third postoperative day (T7).

Other secondary outcomes included maternal anxiety scores (assessed by the Pregnancy-Related Anxiety Questionnaire–Revised 2 [PRAQ-R2] at T2, T4, T6, T7, and the first month post partum [T8], comprising 10 items, each rated on a 5-point Likert scale [score range, 1-5, with higher scores indicative of increased pregnancy-related anxiety]),^28,29^ maternal depression scores (assessed by the Edinburgh Postnatal Depression Scale [EPDS] at T2, T4, T6, T7, and T8, including 10 items, scored on a 4-point scale [score range, 0-3, with higher scores denoting more severe depressive symptoms]),^30^ sleep quality (assessed by the Leeds Sleep Evaluation Questionnaire [LSEQ] at T2, T4, and T6, [score range, 0-100, where higher scores indicate better sleep quality]),^31^ and quality of postpartum recovery (assessed by the 11-item Obstetric Quality Recovery Scale [ObsQoR-11] at T4, T6, and T7 [score range, 0-110, where higher scores indicate better quality of recovery]).^32^ The timeline for this trial is shown in eFigure 2 in Supplement 2.

### Statistical Analysis

Statistical analysis was performed in September 2024. Based on our preliminary observation, the incidence of moderate to severe uterine contraction pain on the third postoperative day after a lower-segment transverse cesarean delivery was 35.0% (7 of 20 patients). Considering that the number needed to treat for a moderate treatment effect for neuropathic pain was 4 to 10,^33,34,35^ we set the number needed to treat at 5 and the absolute risk reduction between the control and the intervention groups at 0.2. Given the absolute risk reduction value of 0.2, we hypothesized that after 3 sessions of the active taVNS, the incidence of moderate to severe uterine contraction pain on the third postoperative day would be reduced to 15%. With a statistical power of 0.80 and α = .05, we calculated that a sample size of 70 patients per group was required using Power Analysis & Sample Size, version 15.0 (NCSS Statistical Software). Assuming a 10% loss to follow-up, we planned to recruit 78 participants per group.

Missing data were largely assumed to be missing at random. There were no missing data for the primary outcome, and missing data for all secondary outcomes were less than 5%, so we did not perform an imputation of missing data. All analyses were conducted according to the intention-to-treat principle.

The comparison of primary outcomes was performed using generalized estimating equations. The treatment × time interaction was tested first. If this interaction was statistically significant, between-group differences were tested at each individual time point with a Bonferroni correction for multiple comparisons. Otherwise, the main effect of treatment was subsequently tested without applying a Bonferroni correction when assessing treatment effects at each time point. For other repeatedly measured data, generalized estimating equations were similarly used for analysis. The χ^2^ test or the Fisher exact test was performed for categorical variables expressed as frequency. Assessment for normal distribution was performed with the Shapiro-Wilk test. Normally distributed data were presented as mean (SD) values and compared by the independent sample *t* test. Nonnormally distributed data were presented as median (IQR) values and compared by the Mann-Whitney test.

In the exploratory analysis, we compared the area under the curve of the VAS scores for peak uterine contraction pain and incisional pain from T1 to T7 postoperatively, and we compared the conditions of moderate to severe incision pain. We also recorded and compared the use of patient-controlled intravenous analgesia solution at 24 and 48 hours postoperatively, the volume of lochia discharge on the first postoperative day, the height of the uterine fundus, the time of catheter removal, the time to first urination after catheter removal, and adverse events during hospitalization. In addition, the difference in the primary outcome was assessed in logistic regression models to determine the treatment-by-covariate interactions for each subgroup factor. Based on the uterine contraction pain score on the third postoperative day, patients were categorized into 2 groups: no to mild pain and moderate to severe pain. Variables with *P* < .20 in univariable analyses were included in the multivariable logistic regression model to control for confounding factors. We used variance inflation factors to examine possible collinearity among covariates and added the group as a relevant variable in the multivariable model. We checked the model fitness for the logistic regression using the Hosmer-Lemeshow goodness-of-fit test statistics. We used the forest plot to show the result of multivariable logistic regression and examined 5 subgroups identified based on differences in bivariate analyses. Data were analyzed using SPSS, version 27.0 (IBM Corp) and GraphPad Prism, version 9 (GraphPad Software), with 2-sided *P* < .05 considered statistically significant.

## Results

### Study Population

A total of 368 parturients scheduled for cesarean delivery under combined spinal-epidural anesthesia were screened. Of these, 212 patients were excluded because they declined participation (72 patients), gestational age outside the 37 to 42 weeks’ range (87 patients), American Society of Anesthesiologists (ASA) physical status classification of IV (2 patients), gemellary pregnancy (12 patients), communication difficulties (3 patients), refusal of postoperative intravenous analgesia pumps (35 patients), and ear skin infection (1 patient). A total of 156 patients, 78 in the active taVNS group (mean [SD] age, 31.5 [4.3] years) and 78 in the sham taVNS group (mean [SD] age, 31.3 [4.5] years), were randomly assigned (Table 1). In the active taVNS group, 3 patients were lost to follow-up after hospital discharge (1 refused postdischarge assessments, and 2 had incorrect follow-up contact details). In the sham taVNS group, 5 patients were lost to follow-up after hospital discharge (2 refused postdischarge assessments, and 3 had incorrect follow-up contact details). The participant flow diagram is presented in the Figure, and the demographic and intraoperative characteristics are detailed in Table 1 and Table 2.

**Table 1.** Baseline Characteristics of Study Participants in the Active taVNS and Sham taVNS Groups

Characteristic	Active taVNS (n = 78)	Sham taVNS (n = 78)	*P* value
Age, mean (SD), y	31.5 (4.3)	31.3 (4.5)	.77
BMI, mean (SD)	29.0 (3.2)	29.0 (3.5)	.97
ASA physical status classification, No. (%)			
II	75 (96.2)	73 (93.6)	.47
III	3 (3.8)	5 (6.4)	
Gestational age, mean (SD), d	272.9 (6.7)	272.3 (6.8)	.61
Multipara, No. (%)			
No	38 (47.4)	39 (48.7)	.87
Yes	40 (52.6)	39 (51.3)	
Gravidity, median (IQR), No.	1 (0-2)	1 (0-2)	.64
Parity, median (IQR), No.	1 (0-1)	0.5 (0-1)	.83
Dysmenorrhea, No. (%)	29 (37.2)	33 (42.3)	.51
Prenatal contractions, No. (%)	40 (51.3)	42 (53.8)	.75
Educational level, No. (%)			
Elementary school	2 (2.6)	2 (2.6)	.86
Middle school	12 (15.4)	13 (16.7)	
Technical secondary school	6 (7.7)	7 (9.0)	
High school	4 (5.1)	2 (2.6)	
Junior college	23 (29.5)	18 (23.1)	
College graduate	30 (38.5)	33 (42.3)	
Master’s degree	1 (1.3)	3 (3.8)	
PSQI score, median (IQR)	8 (6-10)	8 (7-10)	.24

Abbreviations: ASA, American Society of Anesthesiologists; BMI, body mass index (calculated as weight in kilograms divided by height in meters squared); PSQI, Pittsburgh Sleep Quality Index; taVNS, transcutaneous auricular vagus nerve stimulation.

**Figure.**
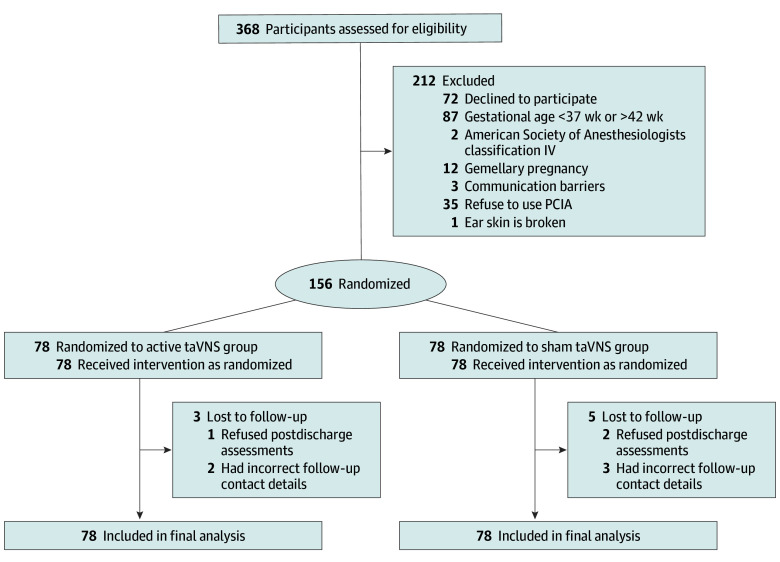
Flow Diagram PCIA indicates patient-controlled intravenous analgesia; taVNS, transcutaneous auricular vagus nerve stimulation.

**Table 2.** Intraoperative and Postoperative Data Between the Active taVNS and Sham taVNS Groups

Characteristic	Active taVNS (n = 78)	Sham taVNS (n = 78)	*P* value
Anesthesia time, median (IQR), min	80.0 (70.0 to 91.3)	80.0 (70.0 to 90.0)	.95
Surgery time, median (IQR), min	65.0 (55.0 to 76.3)	65.0 (55.0 to 75.0)	.95
Duration of delivery, median (IQR), s	277.0 (269.0 to 284.0)	275.5 (268.0 to 283.0)	.46
Level of anesthesia, No. (%)			
Fourth thoracic spine	27 (34.6)	26 (33.3)	.69
Fifth thoracic spine	41 (52.6)	45 (57.7)	
Sixth thoracic spine	10 (12.8)	7 (9.0)	
Lumbar puncture space, No. (%)			
L2-L3	41 (52.6)	45 (57.7)	.52
L3-L4	37 (47.4)	33 (42.3)	
Infusion quantity, median (IQR), mL	1213 (880 to 1213)	1020 (900 to 1250)	.52
Estimated blood loss, median (IQR), mL	390 (358 to 420)	400 (360 to 426)	.52
Urine volume, median (IQR), mL	220 (180 to 270)	200 (170 to 236)	.10
Neonatal weight, mean (SD), g	3424.1 (409.6)	3424.4 (435.0)	1.00
Neonatal sex, No. (%)			
Male	40 (51.3)	44 (56.4)	.52
Female	38 (48.7)	34 (43.6)	
Breastfeeding, No. (%)	52 (66.7)	46 (59.0)	.32
Intraoperative oxytocin, median (IQR), IU	10 (10 to 10)	10 (10 to 10)	.13
Intraoperative uterine stimulating drugs, No. (%)			
Ergometrine	12 (15.4)	9 (11.5)	.71
Carbetocin	32 (41.0)	36 (46.2)	
Postoperative oxytocin, median (IQR), IU			
D0	10 (10 to 10)	10 (10 to 10)	.75
D1	10 (10 to 10)	10 (10 to 10)	.98
D2	10 (10 to 10)	10 (10 to 10)	.18
D3	10 (0 to 10)	0 (0 to 10)	.47
Urinary catheter indwelling time, median (IQR), h	23.0 (21.4 to 25.1)	23.0 (21.0 to 24.6)	.79
Time to first urination after catheter removal, median (IQR), h	2.0 (1.0 to 3.5)	2.5 (2.0 to 4.0)	.03
Lochia volume at 24 h post partum, median (IQR), mL	100 (85 to 115)	110 (90 to 130)	.06
Fundus to the umbilical level at 24 h postpartum, median (IQR), fingerbreadth	−1 (−1 to −1)	−1 (−1 to −1)	.41
Hospitalization duration, mean (SD), day	4.7 (0.9)	4.8 (0.9)	.70

Abbreviations: D0, the day of operation; D1, the first day after operation; D2, the second day after operation; D3, the third day after operation; L2, second lumbar spine; L3, third lumbar spine; L4, fourth lumbar spine; taVNS, transcutaneous auricular vagus nerve stimulation.

### Primary Outcome

The incidence of moderate to severe uterine contraction pain on the third postoperative day (T7) between groups was significantly different (4 of 78 patients [5.1%] in the active taVNS group vs 22 of 78 patients [28.2%] in the sham taVNS group; relative risk, 0.18 [95% CI, 0.07-0.50]; *P* < .001) (Table 3; eFigure 3B in Supplement 2).

**Table 3.** Comparison of Contraction Pain and Incisional Pain Between the Active taVNS and Sham taVNS Groups

Variable	Active taVNS (n = 78)	Sham taVNS (n = 78)	*P* value
**Moderate to severe contraction pain, No. (%)^a^**			
T1	66 (84.6)	70 (89.7)	.34
T2	49 (62.8)^b^	59 (75.6)	.09
T3	66 (84.6)	67 (85.9)	.82
T4	22 (28.2)^b^	36 (46.2)^c^	.02
T5	12 (15.4)^b^	48 (61.5)^c^	<.001
T6	6 (7.7)^b^	21 (26.9)^c^	.003
T7	4 (5.1)^b^	22 (28.2)^c^	<.001
**Contraction pain score, median (IQR)^d^**			
T1	5.6 (3.9-7.0)	5.7 (4.6-7.0)	.40
T2	4.2 (3.0-5.6)^b^	4.6 (3.6-5.5)^c^	.18
T3	5.0 (4.0-6.5)	5.5 (4.2-6.7)	.33
T4	2.7 (2.0-3.6)^b^	3.3 (2.3-4.5)^c^	.04
T5	2.5 (2.0-3.0)^b^	3.9 (3.0-4.8)^c^	<.001
T6	2.0 (1.2-2.5)^b^	2.6 (1.8-3.6)^c^	<.001
T7	2.0 (1.0-2.5)^b^	2.9 (1.9-3.7)^c^	<.001
**Incisional pain score, median (IQR)^e^**			
T1	4.55 (3.78-5.68)	4.60 (4.00-5.60)	.95
T2	3.75 (3.19-4.76)^b^	4.60 (4.00-5.50)	<.001
T3	3.50 (2.88-4.00)^b^	4.25 (3.70-5.00)	<.001
T4	2.75 (2.40-3.31)^b^	4.00 (3.73-5.00)^f^	<.001
T5	2.80 (2.50-3.13)^b^	3.60 (3.00-4.13)^c^	<.001
T6	2.20 (2.00-2.46)^b^	3.50 (3.00-4.00)^c^	<.001
T7	2.20 (2.00-2.50)^b^	3.00 (2.60-3.33)^c^	<.001
AUC of contraction pain, mean (SD)	20.41 (2.44)	24.20 (2.51)	<.001
AUC of incisional pain, mean (SD)	19.01 (1.36)	24.90 (1.69)	<.001
PCIA within 24 h, median (IQR), mL	53 (51-56)	58 (54-62)	<.001
PCIA within 48 h, median (IQR), mL	103 (101-107)	108 (102-113)	<.001

Abbreviations: AUC, area under the curve; PCIA, patient-controlled intravenous analgesia; taVNS, transcutaneous auricular vagus nerve stimulation; T1, before the first taVNS intervention on the day of operation; T2, after the first taVNS intervention on the day of operation; T3, before the second taVNS intervention on the first day after operation; T4, after the second taVNS intervention on the first day after operation; T5, before the third taVNS intervention on the second day after operation; T6, after the third taVNS intervention on the second day after operation; T7, the third day after operation.

^a^ The incidence of moderate to severe uterine contraction pain was compared in the generalized estimated equation model (*P* < .001 for group, *P* < .001 for time, *P* = .002 for treatment-by-time interaction; Wald χ^2^ = 16.52 for group, Wald χ^2^ = 237.80 for time, Wald χ^2^ = 21.32 for interaction).

^b^ *P* < .001 for each time point vs T1 within the active taVNS group.

^c^ *P* < .001 for each time point vs T1 within the sham taVNS group.

^d^ Contraction pain was compared in the generalized estimated equation model (*P* < .001 for group, *P* < .001 for time, *P* < .001 for treatment-by-time interaction; Wald χ^2^ = 12.88 for group, Wald χ^2^ = 1037.69 for time, Wald χ^2^ = 49.94 for interaction).

^e^ Incisional pain scores were compared in the generalized estimated equation model between groups (*P* < .001 for group, *P* < .001 for time, *P* < .001 for interaction; Wald χ^2^ = 68.94 for groups, Wald χ^2^ = 828.95 for time, Wald χ^2^ = 374.06 for interaction).

^f^ *P* = .01 for each time point vs T1 within the sham taVNS group.

### Secondary Outcomes

Contraction pain scores in the active taVNS group generally decreased, whereas in the sham taVNS group, contraction pain scores showed a fluctuating trend overall (eFigure 3A in Supplement 2). Incision pain scores in both groups generally showed a decreasing trend (eFigure 3C in Supplement 2). The active taVNS group exhibited a significant difference in incision pain scores compared with the sham taVNS group starting from the first intervention (T2). The median incision pain score at T7 was 2.20 (IQR, 2.00-2.50) in the active taVNS group compared with 3.00 (IQR, 2.60-3.33) in the sham taVNS group (*P* < .001) (Table 3). The area under the curve of uterine contraction pain (mean [SD], 20.41 [2.44] vs 24.20 [2.51]; *P* < .001) and area under the curve of incision pain (mean [SD], 19.01 [1.36] vs 24.90 [1.69]; *P* < .001) from T1 to T7 in the active taVNS group were significantly lower than those in the sham taVNS group (Table 3; eFigure 4 in Supplement 2).

The active taVNS group had higher median LSEQ scores compared with the sham taVNS group (T6: 52.00 [IQR, 50.00-55.00] vs 47.50 [IQR, 43.00-52.00]) indicating better sleep, and higher median ObsQoR-11 scores (T7: 104 [IQR, 103-105] vs 99 [IQR, 96-101]), indicating better postoperative recovery (Table 4; eFigure 5 in Supplement 2). In addition, the active taVNS group had lower median EPDS scores than the sham taVNS group (T7: 3.00 [IQR, 2.00-4.00] vs 5.00 [IQR, 3.00-6.00]), suggesting relief of depression, and lower median PRAQ-R2 scores (T7: 13.50 [IQR, 12.00-15.00] vs 15.00 [IQR, 13.75-17.00]), suggesting relief of anxiety (Table 4; eFigure 5 in Supplement 2).

**Table 4.** Comparison of Other Outcomes Between the Active taVNS and Sham taVNS Groups

Characteristic	Active taVNS (n = 78)	Sham taVNS (n = 78)	*P* value
**EPDS score, median (IQR)^a^**			
T0	7.50 (5.00-9.00)	7.00 (5.00-10.00)	.70
T2	6.50 (5.00-8.00)	8.00 (6.00-10.25)	.001
T4	5.00 (4.00-6.00)	6.00 (4.75-8.00)	.002
T6	4.00 (3.00-5.00)	5.00 (3.00-7.00)	<.001
T7	3.00 (2.00-4.00)	5.00 (3.00-6.00)	<.001
T8	3.00 (2.00-4.00)	3.00 (3.00-4.00)	.53
**PRAQ-R2 score, median (IQR)^a^**			
T0	24.50 (21.00-28.00)	25.50 (20.00-30.00)	.34
T2	18.00 (15.00-20.00)	19.00 (17.00-23.00)	<.001
T4	17.00 (15.00-19.00)	18.00 (16.00-20.00)	.002
T6	15.00 (13.00-16.00)	16.00 (15.00-18.25)	<.001
T7	13.50 (12.00-15.00)	15.00 (13.75-17.00)	<.001
T8	13.00 (12.00-14.00)	14.00 (13.00-15.00)	.20
**LSEQ score, median (IQR)^a^**			
T0	42.00 (38.00-49.25)	43.00 (40.75-47.00)	.83
T2	43.00 (39.75-46.00)	39.00 (35.00-42.00)	<.001
T4	49.00 (45.00-52.00)	46.00 (41.00-49.00)	.001
T6	52.00 (50.00-55.00)	47.50 (43.00-52.00)	<.001
**ObsQoR-11 score, median (IQR)^a^**			
T0	105 (102-106)	105 (103-106)	.92
T4	85 (83-87)	83 (80-86)	.006
T6	96 (94-98)	92 (89-94)	<.001
T7	104 (103-105)	99 (96-101)	<.001
Adverse events, No. (%)			
Headache	2 (2.6)	1 (1.3)	.77
Pain to auricular region	3 (3.8)	1 (1.3)	
Emesis	0	1 (1.3)	
Fever	2 (2.6)	3 (3.8)	
Indwelling time of urinary catheter >24 h	1 (1.3)	1 (1.3)	

Abbreviations: EPDS, Edinburgh Postnatal Depression Scale; LSEQ, Leeds Sleep Evaluation Questionnaire; ObsQoR-11, Obstetric Quality-of-Recovery Score; PRAQ-R2, Pregnancy Anxiety Questionnaire–Revised 2; taVNS, transcutaneous auricular vagus nerve stimulation; T0, 1 day before operation; T2, after the first taVNS intervention on the day of operation; T4, after the second taVNS intervention on the first day after operation; T6, after the third taVNS intervention on the second day after operation; T7, the third day after operation; T8, 1 month after operation.

^a^ The EPDS, PRAQ-R2, LSEQ, and ObsQoR-11 scores were compared in the generalized estimated equation model between groups (*P* < .01 for group, *P* < .001 for time, *P* < .001 for interaction).

To explore the risk factors for moderate to severe uterine contraction pain on the third postoperative day, univariable analyses showed that the treatment group *P* value, along with the *P* values for group, number of deliveries, dysmenorrhea history, postpartum breastfeeding status, uterine contraction pain at T1, and preoperative anxiety at T0, were all less than .20 and were subsequently included in the multivariable logistic regression model. The Hosmer-Lemeshow test yielded a χ^2^ value of 11.43 (*df* = 8; *P* = .18), indicating a good model fit. As shown in the eTable in Supplement 2, taVNS intervention and parity were associated with moderate to severe uterine contraction pain at T7. The interaction terms from the subgroup analysis, used to assess the intervention’s effect, are visualized in the forest plot in eFigure 6 in Supplement 2. The taVNS intervention may be more effective in alleviating postpartum uterine contraction pain among women who have delivered previously (odds ratio, 0.10 [95% CI, 0.03-0.36]; *P* = .03) (eFigure 6 in Supplement 2).

The adverse event of the taVNS intervention was skin tingling in the ear, and patients described it as transient and tolerable. While 2.6% of patients (4 of 156) experienced transient and tolerable paresthesia at the stimulation site, no severe adverse events were reported in either group (Table 4).

## Discussion

This prospective randomized clinical trial demonstrated that 3 sessions of taVNS significantly reduced uterine contraction pain scores and incision pain scores during the 3 days postoperatively among women undergoing elective cesarean delivery. Moreover, the study revealed that active taVNS not only significantly mitigated postoperative pain but also decreased the need for analgesics, alleviated anxiety and depressive symptoms, improved sleep quality, and enhanced overall recovery among postpartum women.

Postpartum uterine contraction pain is influenced by the administration of oxytocin and endogenous oxytocin release during breastfeeding, introducing variability in the timing and intensity of pain episodes. To better understand the effect of taVNS on uterine contraction pain, we differentiated postpartum pain into distinct categories, specifically assessing uterine contraction pain and incision pain separately. Transcutaneous auricular vagus nerve stimulation may alleviate contraction pain by activating key components of the endogenous pain descending modulation system, such as the ventral tegmental area, nucleus of the solitary tract, and raphe nuclei, thereby modulating pain transmission and reducing pain perception while also influencing emotional responses.^36,37^ Our findings are consistent with the study by Patel et al,^38^ which demonstrated that taVNS intervention effectively reduced postoperative pain. We observed that, with increased frequency of taVNS, uterine contraction pain was significantly improved in the active taVNS group compared with the sham taVNS group. After the second intervention, the differences became statistically significant, and even during subsequent oxytocin administration and breastfeeding, the incidence of moderate to severe uterine contraction pain and pain intensity continued to decrease. Previous studies have indicated that taVNS provides sustained analgesic effects while alleviating immediate pain.^39^ Therefore, we conducted 3 sessions of taVNS to harness the cumulative effects of taVNS in mitigating pain perception, thereby optimizing pain management and preventing the onset of complications, which is essential for achieving favorable clinical outcomes.^40^

Compared with women undergoing vaginal delivery, women undergoing cesarean delivery also experience pain from surgical trauma. Approximately 10% of cases involving severe acute postoperative pain may progress to chronic pain, underscoring the critical importance of early interventions to mitigate the persistence and progression of acute pain.^41^ Our findings demonstrate that incision pain in the active taVNS group exhibited a significant reduction after the initial intervention, with continued decreases in pain scores. Extensive research has confirmed that early postoperative taVNS can modulate inflammation via the parasympathetic anti-inflammatory pathway, reducing the release of inflammatory factors, such as tumor necrosis factor, interleukin 1β (IL-1β), and IL-6, associated with nerve injury and central sensitization. This modulation helps prevent the development of chronic neuropathic pain.^42,43^

Perinatal depression represents a prevalent complication during pregnancy, with 60% of affected women exhibiting comorbid psychiatric disorders, predominantly anxiety disorders in more than 80% of cases.^44^ Traditional analgesic methods, such as opioid medications, have shown limited efficacy in improving postpartum mental health; in addition, concerns regarding the transfer of medication into breast milk further contribute to maternal psychological distress.^45^ Our study demonstrated that taVNS administered post partum effectively alleviated maternal anxiety and depression. This effect may be attributed to increased extracellular norepinephrine levels in the prefrontal cortex and hippocampus, as well as upregulation of 5-HT_1B_ receptors on hippocampal neurons, thereby modulating perioperative depressive symptoms and mitigating anxiety and depression.^46^ Perinatal anxiety and depression are chronic conditions with long-term implications. It is crucial to evaluate the sustained effect of the postsurgical period.^47^ The long-term effects of taVNS on neuroplasticity may yield sustained therapeutic benefits.^48^ This study found that taVNS alleviated symptoms of maternal anxiety and depression during hospitalization; however, no significant differences in anxiety or depression levels were observed at the 1-month follow-up. This phenomenon may be attributed to fluctuations in postpartum hormone levels and the influence of family-related factors.

The study also found that taVNS enhanced postoperative sleep quality in the early postpartum period. Zhang et al^49^ and Luo et al^50^ proposed that taVNS may improve sleep by alleviating anxiety and depression, as well as by modulating functional connectivity within the solitary tract nucleus–corneal brain network and default mode network. In our study, taVNS was also found to enhance recovery quality, potentially due to its effects on pain relief, sleep improvement, and emotional regulation.

The safety of taVNS was also evaluated in this study. A comprehensive meta-analysis indicated that no causal link was established between taVNS and severe adverse events.^51^ These findings are consistent with our study, in which no significant treatment-related adverse events were observed, although 2.6% of patients experienced transient and tolerable paresthesia at the stimulation site. Other instances of headache were attributed to complications associated with spinal anesthesia. Collectively, these data suggest that taVNS is a safe and viable clinical intervention for alleviating postpartum uterine contraction pain among women undergoing cesarean delivery.

### Limitations

This study has several limitations that warrant consideration. First, as a single-center trial, the study focused exclusively on women undergoing elective cesarean delivery receiving combined spinal-epidural anesthesia, which may limit the generalizability and external validity of the findings. Second, this trial did not incorporate objective measures, such as biomarkers (eg, inflammatory cytokines), nor did it assess parameters such as heart rate variability, thereby limiting the ability to conduct a comprehensive and objective evaluation of the intervention’s effect. Third, this study did not evaluate the effectiveness of blinding by asking participants to guess the type of stimulation they received after the trial, so as to reduce subjective bias and ensure the objectivity of trial results. Fourth, there is uncertainty regarding the optimal stimulation frequency and duration of the intervention. A significant limitation is the inability to definitively determine whether customizing the frequency and duration of the intervention is necessary to achieve an optimal clinical effect, which may vary based on individual differences in the autonomic nervous system of the parturients.

## Conclusions

In this randomized clinical trial involving women undergoing cesarean delivery, our findings indicate that 3 sessions of taVNS substantially reduced the incidence of moderate to severe postpartum uterine contraction pain. Furthermore, taVNS significantly improved perioperative anxiety and depression scores, enhanced sleep quality, and optimized recovery outcomes. These results suggest that taVNS represents a novel and safe approach for alleviating postpartum uterine contraction pain in women after cesarean delivery, and it may be considered as a routine adjunctive treatment for pain management in patients who have undergone cesarean delivery.

## Notes

Supplement 1.
Trial Protocol and Statistical Analysis Plan

Supplement 2. **eTable.** Binary Logistic Regression Analysis to Identify Moderate to Severe Contraction Pain at T7 **eFigure 1.** Apparatus Used in This Test **eFigure 2.** Timeline of the Experimental Design of the Two Experimental Sessions (Active-taVNS and Sham-taVNS) **eFigure 3.** (A) Comparison of Contraction Pain Scores Between Groups; (B) Comparison of Contraction Pain Intensity Between Groups; (C) Comparison of Incisional Pain Scores Between Groups; (D) Comparison of Incisional Pain Intensity Between Groups **eFigure 4.** (A)(B) Comparison of AUC of Contraction Pain in Time Periods T1-T7 Between Groups; (C)(D) Comparison of AUC of Incisional Pain in Time Periods T1-T7 Between Groups **eFigure 5.** Comparison of EPDS, PRAQ-R2, LSEQ and ObsQoR-11 **eFigure 6.** Forest Plot of the Subgroup Analysis

Supplement 3.
Data Sharing Statement
