# Supplementary material for: Transcutaneous Auricular Vagus Nerve Stimulation for Postpartum Contraction Pain During Elective Cesarean Delivery: A Randomized Clinical Trial
Source: JAMA Netw Open. 2025 Aug 29;8(8):e2529127. doi: 10.1001/jamanetworkopen.2025.29127 (PMC12397895; doi:10.1001/jamanetworkopen.2025.29127)
Supplement: Supplement 1. — Trial Protocol and Statistical Analysis Plan [file jamanetwopen-e2529127-s001.pdf]

Transcutaneous Auricular Vagus Nerve Stimulation for Postpartum Contraction Pain During Elective Cesarean Delivery：A Randomized Clinical Trial

Study Protocol

Principal Investigator: Xingyu Xiong, MD, Yanyu Jiang, MD, Song Zhang, PhD, He Liu, PhD, Jun-Li Cao, PhD.

Protocol Synopsis

**Title:**	Transcutaneous Auricular Vagus Nerve Stimulation for Postpartum Contraction Pain During Elective Cesarean Delivery: A Randomized Clinical Trial
**Study Type:**	A prospective, single-center, randomized, double-blind clinical trial
**Corresponding Author:**	Jun-Li Cao, Department of Anesthesiology, The Affiliated Hospital of Xuzhou Medical University, Xuzhou, China. NMPA Key Laboratory for Research and Evaluation of Narcotic and Psychotropic Drugs & Jiangsu Province Key Laboratory of Anesthesiology & Jiangsu Province Key Laboratory of Anesthesia and Analgesia Application Technology, Xuzhou Medical University, Xuzhou 221004, Jiangsu, China.<br>He Liu, Department of Anesthesiology & Clinical Research Center for Anesthesia and Perioperative Medicine & Key

	Laboratory of Anesthesia and Analgesia Application Technology, Huzhou Central Hospital, The Fifth School of Clinical Medicine of Zhejiang Chinese Medical University || Huzhou Central Hospital, The Affiliated Central Hospital Huzhou University || Affiliated Huzhou Hospital, Zhejiang University School of Medicine, Huzhou 313003, Zhejiang, China.
**Study Centers:**	The Affiliated Hospital of Xuzhou Medical University
**Ethics**	Approved by the Medical Ethics Committee of the Affiliated Hospital of Xuzhou Medical University (XYFY2024-KL053-02)
**Trial Registration:**	www.chictr.org.cn Identifiers: ChiCTR2400082716

1. Study Objectives

Postpartum uterine contraction pain is a severe, episodic lower abdominal discomfort experienced by women during the early postpartum period as a result of uterine contraction. Transcutaneous auricular vagus nerve stimulation (taVNS) has demonstrated promising efficacy in the management of both acute and chronic pain conditions.

The objective of this study is to evaluate the efficacy of taVNS in alleviating postpartum uterine contraction pain in elective cesarean section women under combined spinal-epidural anesthesia.

2. Background

Cesarean delivery is a critical, life-saving surgical procedure utilized in situations such as prolonged labor, obstructed labor, fetal distress, or abnormal fetal positioning[1]. In addition to the common short-term and long-term risks associated with surgical procedures, women undergoing cesarean delivery also experience postpartum uterine contraction pain comparable to that experienced by women who deliver vaginally[2][3]. Postpartum uterine contraction pain typically emerges within 1 to 2 days following delivery and is characterized by intermittent and intense lower abdominal discomfort[4]. The frequent and intense nature of uterine contraction is closely related to perioperative anxiety, depression, and sleep disturbances, which can adversely affect the quality of postpartum recovery and pose significant risks to the mother's physical and psychological well-being[5][6]. Therefore, it is imperative to address and mitigate the pain associated with uterine contraction following cesarean delivery surgery.

Currently, the most commonly employed analgesic methods include epidural analgesia and intravenous administration of analgesics. While all of these methods are effective, they all have different side effects, including drowsiness, nausea, vomiting, itching, constipation, and respiratory depression[7]. Moreover, these medications may impact the breastfed neonate, thereby imposing an additional psychological burden on the mother during lactation[8].

Transcutaneous auricular vagus nerve stimulation (taVNS) is a noninvasive neuromodulation technique that delivers low-intensity electrical current through electrodes to stimulate the auricular branch of the vagus nerve. taVNS exerts analgesic effects by modulating the vagus nerve system and its projections to pain-specific brain nuclei[9][10], and has also been shown to alleviate anxiety and depression in patients[11][12]. However, there is limited research on the impact of taVNS on postpartum uterine contraction pain. Therefore, this study aims to investigate the analgesic effect of taVNS on postpartum uterine contraction pain and to explore its potential in promoting postpartum recovery in women under combined spinal-epidural anesthesia for elective cesarean delivery.

3. Methods

This randomized, double-blind, controlled trial is conducted at the Department of Anesthesiology and Obstetrics of the Affiliated Hospital of Xuzhou Medical University. The study protocol received approval from the Ethics Committee of the Affiliated Hospital of Xuzhou Medical University (approval number: XYFY2024-KL053-02) and registered with the China Clinical Trial Registry on April 6, 2024 (ChiCTR2400082716). This report follows the Consolidated Standards of Reporting Trials (CONSORT) guidelines for randomized studies.

3.1 Recruiting Methods and Consent Procedure

The day prior to surgery, potential participants are identified from the women

scheduled for elective cesarean section. All potential participants who meet the inclusion criteria as defined by the chief anesthesiologist and designated personnel are invited to participate in the study. During the screening phase, participants or their legal surrogates are provided with the informed consent form, allowing them the opportunity to thoroughly review the document, ask any questions, and seek clarification on any aspect of the study. Prior to signing the consent form, the researchers address further inquiries from the participants. Once the consent form is signed, the participant receive a copy of the signed and dated form, with another copy placed in the medical records at the Affiliated Hospital of Xuzhou Medical University.

3.2 Inclusion Criteria and Exclusion Criteria

Inclusion criteria include women with a singleton pregnancy at 37–42 weeks of gestation; aged 18 years or older; classified as American Society of Anesthesiologists (ASA) physical status classification of II or III; scheduled to undergo a cesarean delivery via a lower segment transverse incision under combined spinal-epidural anesthesia. Exclusion criteria include refusal to sign the informed consent form, a history of neurological or psychiatric disorders, cognitive impairment or communication difficulties, auricular dermatitis, substance abuse, or an inability to use patient-controlled intravenous analgesia. Participants may withdraw from the study for reasons such as voluntary withdrawal, poor adherence, protocol deviations, or failure to complete follow-up.

3.3 Randomization and Blinding

Participants are randomly assigned in a 1:1 ratio to either the active taVNS group or the sham taVNS group based on a computer-generated random number sequence. The allocation information is concealed in opaque envelopes and remains concealed until participants are ready to initiate the first intervention in the ward. All personnel involved in data collection, processing, and analysis, as well as other healthcare staff and participants, are blinded to the group assignments. Blinding will be disclosed after statistical analysis of the trial results.

3.4 Follow-up Plan

On the day before the parturient's surgery (T0), researchers will collect baseline data, and assess sleep at T0 on the morning of the surgery. On the day of the surgery, before the first intervention (T1), blinded researchers will evaluate the peak uterine contraction pain and incision pain. After the first intervention (T2), depression and anxiety will be assessed, followed by re-evaluation of peak uterine contraction pain and incision pain during this period. Sleep at T2 will be assessed on the morning of the first postoperative day. Before the second intervention (T3), peak uterine contraction pain and incision pain will be evaluated. After the second intervention (T4), depression, anxiety, postpartum recovery quality, and peak uterine contraction pain and incision pain during this period will be assessed. Sleep at T4 will be evaluated on the morning of the second postoperative day. Before the third intervention

(T5), peak uterine contraction pain and incision pain will be evaluated. After the third intervention (T6), depression, anxiety, postpartum recovery quality, and peak uterine contraction pain and incision pain during this period will be assessed. On the morning of the third postoperative day (T7), sleep at T6 will be evaluated, along with peak uterine contraction pain, incision pain, depression, anxiety, and postpartum recovery quality. At 1 month postpartum (T8), the telephone follow-up will assess the parturient's depressive and anxiety status.

3.5 Outcome measures

3.5.1 Primary outcome

The primary outcome is the incidence of moderate to severe uterine contraction pain on the third postoperative day. Pain intensity is evaluated using the Visual Analogue Scale (VAS). The VAS is a well-established tool for evaluating pain, consisting of a 100 mm line where one end represents "no pain" and the other end signifies "the worst pain imaginable" or "the most extreme pain possible"[13]. Participants are asked to mark the point on the line that best corresponded to their perceived pain intensity. In this study, uterine contraction pain is classified as moderate to severe if the VAS score is 4 or higher.

3.5.2 Secondary outcomes

1) Peak uterine contraction pain scores during the three days postoperatively (assessed by the VAS). The contraction pain scores are measured before the

first intervention (T1), after the first intervention (T2), before the second intervention (T3), after the second intervention (T4), before the third intervention (T5), after the third intervention (T6), and the third postoperative day (T7).

2) Incision pain scores at T1-T7 (assessed by the VAS).

3) Maternal anxiety scores at T2, T4, T6, T7 and the first month postpartum [T8] (assessed by the Pregnancy-Related Anxiety Questionnaire-Revised 2 [PRAQ-R2], comprising 10 items, each rated on a 5-point scale [1-5], with higher scores indicative of increased pregnancy-related anxiety)[14].

4) Maternal depression scores at T2, T4, T6, T7, and T8 (assessed by the Edinburgh Postnatal Depression Scale [EPDS], including 10 items, scored on a 4-point scale [0-3], with higher scores denote more severe depressive symptoms)[15].

5) Sleep quality at T2, T4 and T6 (assessed by the Leeds Sleep Evaluation Questionnaire [LSEQ], with higher scores indicating better sleep quality)[16].

6) Quality of postpartum recovery at T4, T6 and T7 (assessed by the 11-item Obstetric Quality Recovery Scale [ObsQoR-11], with a total score range of 0-110)[17].

3.5.3 Adverse Events

Adverse events (AE) refer to unforeseen medical occurrences experienced by clinical study participants. Adverse events are typically mild discomforts, with the most common being ear pain, headache, and tingling sensations. Some

patients may experience mild headaches or localized tingling. In this study, investigators will observe participants' facial expressions and verbal responses when they first receive stimulation, avoiding leading questions (such as inquiring whether the participant experiences tingling or discomfort).

3.6 Statistical Analysis

Descriptive statistics will include the Chi-squared test or Fisher exact test for categorical variables expressed as frequency. Assessment for normal distribution will be performed with the Shapiro–Wilk test. Normally distributed data will be presented as mean and standard deviation (SD) and compared by the independent sample t-test. Non-normally distributed data will be presented as median and interquartile range (IQR) and compared by the Mann-Whitney U test.

For data collected at multiple time points, we will adopt the Generalized Estimating Equations (GEE). First, we will test the interaction between treatment and time. If this interaction is significant, we will test the between-group differences at each time point and use the Bonferroni test to adjust for multiple comparisons. Otherwise, we will next test the main effect of treatment, and the Bonferroni correction will not be performed for the treatment effects at each time point. For the comparison of the incidence rate of moderate to severe postpartum contraction pain, the GEE model will include the following settings: covariates: intervention group, time, intervention × time interaction; link function: logit function; group variable: participant; intraclass

correlation structure: independent. The GEE models for contraction pain score, incision pain score, depression score, anxiety score, sleep score, and recovery quality score will include the following settings: covariates: intervention group, time, intervention × time interaction; link function: identity link; group variable: participant; intraclass correlation structure: independent. Data will be analyzed using SPSS version 27.0 (IBM Corp., Chicago, ILUSA) and GraphPad Prism V.9 (GraphPad Software, San Diego, CAUSA), with *P* <.05 consideredstatistically significant.

3.7 Sample Size Calculation

Based on our preliminary observation, the incidence of moderate to severe uterine contraction pain on the third postoperative day following a lower-segment transverse cesarean delivery is approximately 35% (7 out of 20 patients). Considering that the number needed to treat for a moderate treatment effect for neuropathic pain is 4-10[18][19][20], we set the NNT at 5 and the absolute risk reduction (ARR) between the control and the intervention groups as 0.2. Given the ARR value of 0.2, we hypothesized that after three sessions of the active taVNS, the incidence of moderate to severe uterine contraction pain on the third postoperative day would be reduced to 15%. The sample size calculation for comparing two proportions was performed using the Z-Test with Unpooled Variance. The power calculation employed the Normal Approximation method, with a Two-Sided Alternative Hypothesis specified to assess statistical significance of the proportional difference. The

test type was defined as Unpooled Z-Test to accommodate potential between-group variance heterogeneity. A strategy of equal sample allocation (N1 = N2) was adopted, with input parameters specified as proportions. With a statistical power of 0.80 and α = 0.05, we calculated that a sample size of 70 patients per group was required using PASS version 15.0 (NCSS). Assuming a 10% loss to follow-up, we planned to recruit 78 participants per group.[21][22][23[24][25]

3.8 Intervention with taVNS

On the day of delivery, the first postpartum day, and the second postpartum day, the intervention group received three sessions of active taVNS in total, and the control group received three sessions of sham taVNS in total. Electrical stimulation is applied through electrodes placed on the left concha of the ear (tVNS501; Changzhou Ruishen'an Medical Devices Co., Ltd.). In the active taVNS group, participants receive taVNS (pulse width: 200 μs; frequency: 20 Hz) for 30 minutes, with the current increasing for 30 seconds until the participant reports a tingling sensation, then decreasing to a level slightly below this threshold for 30 minutes. In the sham taVNS group, electrodes are positioned similarly, with the current increasing for 30 seconds until the participant reported a tingling sensation, then decreasing to a level slightly below this threshold, after which the device remained off for the following 30 minutes to simulate the sham state. Throughout the intervention, researchers closely monitor participants for any adverse effects and stop the

stimulation immediately if intolerable side effects are observed.

3.9 Anesthesia protocol

Upon entering the operating room, a peripheral intravenous line is established, and standard monitoring of blood pressure, electrocardiogram, and pulse oximetry is initiated. Following skin disinfection, anesthesiologists perform combined spinal-epidural anesthesia after administering a local infiltration of 2% lidocaine. The lumbar puncture is conducted at the Lumbar spine 2 (L2) - L3 or L3 - L4 intervertebral spaces. For the intrathecal administration, 1.0-1.5 ml of 0.75% bupivacaine hydrochloride is intrathecally injected. Subsequently, the spinal needle is removed, and the epidural catheter is advanced 3-5 cm in the cephalad direction within the epidural space. If necessary, an additional 5 ml of 2% lidocaine is administered via the epidural catheter to provide supplemental analgesia. The maximum level of anesthesia is maintained at thoracic spine 4 - thoracic spine 6. Throughout the procedure, oxygen is delivered via a nasal cannula at a flow rate of 2 L/min. Following the delivery of the fetus, intravenous administrations of 0.075 mg palonosetron and 10 mg nalbuphine hydrochloride are performed. Vasopressor agents are administered as required to ensure hemodynamic stability during anesthesia. Postoperative analgesia is administered via a patient-controlled intravenous analgesia pump. The pump is loaded with sufentanil 1.5 μg/kg, nalbuphine 30 mg, and palonosetron 0.15 mg, which are diluted to 120 ml with normal saline. The Patient-Controlled Intravenous Analgesia pump have a loading dose of 2

ml, a basal infusion rate of 2 ml/h, and a self-controlled analgesic dose of 0.5 ml.

4. Date and Safety Monitoring

The clinical study will implement a comprehensive safety monitoring plan based on a thorough risk assessment. Throughout the study, all adverse events (AEs) will be meticulously documented, promptly addressed, and continuously monitored until resolution or stabilization of the patient's condition. Serious adverse events (SAEs) and unexpected incidents will be reported immediately to the ethics committee, regulatory authorities, and drug oversight agencies as required by the protocol. The principal investigator will conduct periodic comprehensive reviews of all AEs and, if necessary, convene an investigator's meeting to evaluate the risks and benefits associated with the study. This trial is designed as a double-blind study, and blinding will be broken only when necessary to ensure participant safety and rights. Independent data monitoring personnel will be assigned to continuously oversee the study data. For high-risk studies, an independent Data Safety Monitoring Board (DSMB) will be established to review accumulated safety and efficacy data and determine whether the study should proceed.

5. Funding

This study is supported in part by grants from the National Key R&D Program

of China (2022ZD0206200 to SZ); the Key Technologies R&D Program of Guangdong Province (2023B0303020003 to J-LC); Jiangsu Basic Research Programs (BK20243035 to J-LC); National Natural Science Foundation of China (81720108013, 82293641, 82130033 to J-LC; 82171227 and 81300957 to HL; 81771453 and 31970937 to HXZ; 82371536 to SZ); Zhejiang Provincial Natural Science Foundation (LY22H090019 to HL); open project fund of the Key Laboratory for NeuroInformation of Ministry of Education (202311KFY00102 to HL); the Natural Science Foundation of Shanghai (21ZR1411300 to YH); and Shenkang Clinical Study Foundation of Shanghai (SHDC2020CR4061 to YH).

6. Information confidentiality

Medical records are securely kept at the hospital, and access will be granted solely to authorized investigators and members of the ethics committees. Any public dissemination of the study results will ensure that patient identities remain confidential and anonymous.

7. References

[1] Sandall J, Tribe RM, Avery L, et al. Short-term and long-term effects of caesarean section on the health of women and children. Lancet. 2018;392(10155):1349-1357. doi: 10.1016/S0140-6736(18)31930-5.

[2] Lavender T, Hofmeyr GJ, Neilson JP, Kingdon C, Gyte GM. Caesarean section for non-medical reasons at term. Cochrane Database Syst Rev. 2006 Jul 19;(3):CD004660. doi: 10.1002/14651858.CD004660.

pub2. Update in: Cochrane Database Syst Rev. 2012;(3):CD004660. doi: 10.1002/14651858.CD004660.pub3.

[3] Mo X, Zhao T, Chen J, et al. Programmed Intermittent Epidural Bolus in Comparison with Continuous Epidural Infusion for Uterine Contraction Pain Relief After Cesarean Section: A Randomized, Double-Blind Clinical Trial. Drug Des Devel Ther. 2022;16:999-1009. doi: 10.2147/DDDT.S350418.

[4] Fang X, Huan Y, Tao Y, et al. Incidence, severity, and determinants of uterine contraction pain after vaginal delivery: a prospective observational study. Int J Obstet Anesth. 2021;46:102961. doi: 10.1016/j.ijoa.2021.102961.

[5] Lavand'homme P. Postoperative cesarean pain: real but is it preventable? Curr Opin Anaesthesiol. 2018;31(3):262-267. doi: 10.1097/ACO.0000000000000585.

[6] Wigert H, Nilsson C, Dencker A, et al. Women's experiences of fear of childbirth: a metasynthesis of qualitative studies. Int J Qual Stud Health Well-being. 2020;15(1):1704484. doi: 10.1080/17482631.2019.1704484.

[7] Richebé P, Capdevila X, Rivat C. Persistent Postsurgical Pain: Pathophysiology and Preventative Pharmacologic Considerations. Anesthesiology. 2018;129(3):590-607. doi:10.1097/ALN.0000000000002238.

[8] Fischer A, Ortner C, Hartmann T, Jochberger S, Klein KU. Welche Medikamente sind in der Stillzeit erlaubt? : Eine Übersicht für den Anästhesisten, Geburtshelfer und Kinderarzt [Which medications are safe while breastfeeding? : A synopsis for the anesthetist, obstetrician and pediatrician]. Wien Med Wochenschr. 2019;169(3-4):45-55. German. doi: 10.1007/s10354-018-0637-z.

[9] Shi Y, Wu W. Multimodal non-invasive non-pharmacological therapies for chronic pain: mechanisms and progress. BMC Med. 2023;21(1):372. doi: 10.1186/s12916-023-03076-2.

[10] Kaniusas E, Kampusch S, Tittgemeyer M, et al. Current Directions in the Auricular Vagus Nerve Stimulation I - A Physiological Perspective. Front Neurosci. 2019;13:854. doi: 10.3389/fnins.2019.00854.

[11] Zhou Q, Yu L, Yin C, et al. Effect of Transauricular Vagus Nerve S

timulation on Rebound Pain After Ropivacaine Single Injection Femoral Nerve Block for Anterior Cruciate Ligament Reconstruction: A Randomized Controlled Trial. J Pain Res. 2022;15:1949-1958. doi: 10.2147/JPR.S370589.

[12] Liu CH, Yang MH, Zhang GZ, et al. Neural networks and the anti-inflammatory effect of transcutaneous auricular vagus nerve stimulation in depression. J Neuroinflammation. 2020;17(1):54. doi: 10.1186/s12974-020-01732-5.

[13] Yang H, Shi W, Fan J, et al. Transcutaneous Auricular Vagus Nerve Stimulation (ta-VNS) for Treatment of Drug-Resistant Epilepsy: A Randomized, Double-Blind Clinical Trial. Neurotherapeutics. 2023;20(3):870-880. doi: 10.1007/s13311-023-01353-9.

[14] Moonesinghe SR, Jackson AIR, Boney O, et al. Standardised Endpoints in Perioperative Medicine-Core Outcome Measures in Perioperative and Anaesthetic Care (StEP-COMPAC) Group. Systematic review and consensus definitions for the Standardised Endpoints in Perioperative Medicine initiative: patient-centred outcomes. Br J Anaesth. 2019;123(5):664-670. doi: 10.1016/j.bja.2019.07.020.

[15] Tendais I, Costa R, Conde A, Figueiredo B. Screening for depression and anxiety disorders from pregnancy to postpartum with the EPDS and STAI. Span J Psychol. 2014;17:E7. doi: 10.1017/sjp.2014.7.

[16] Tarrasch R, Laudon M, Zisapel N. Cross-cultural validation of the Leeds sleep evaluation questionnaire (LSEQ) in insomnia patients. Hum Psychopharmacol. 2003;18(8):603-10. doi: 10.1002/hup.534.

[17] Kumar S, Ashok V, Jain D, Arora A, Singh A, Sikka P. Validation of an obstetric quality of recovery scoring tool (ObsQoR-11) after elective caesarean delivery in a developing country: a prospective observational study. Int J Obstet Anesth. 2022;49:103235. doi: 10.1016/j.ijoa.2021.103235.

[18] Derry S, Wiffen PJ, Kalso EA, et al. Topical analgesics for acute and chronic pain in adults - an overview of Cochrane Reviews. Cochrane Database Syst Rev. 2017;5(5):CD008609. Published 2017 May 12. doi:10.1002/14651858.CD008609.pub2

[19] Finnerup NB, Attal N, Haroutounian S, et al. Pharmacotherapy for n

europathic pain in adults: a systematic review and meta-analysis. Lancet Neurol. 2015;14(2):162-173. doi:10.1016/S1474-4422(14)70251-0

[20] Maguire T, Roy YM, Tyrrell L. Non-prescription (OTC) oral analgesics for acute pain - an overview of Cochrane reviews. Cochrane Database Syst Rev. 2015;2015(11):CD010794. Published 2015 Nov 4. doi:10.1002/14651858.CD010794.pub2

[21] Chow SC, Wang H, Shao J. Sample Size Calculations in Clinical Research (2nd ed.). Chapman and Hall/CRC.

[22] D'Agostino RB, Chase W, Belanger A. The Appropriateness of Some Common Procedures for Testing the Equality of Two Independent Binomial Populations. The American Statistician. 1988;42(3):198-202.

[23] Fleiss JL, Levin B, Paik MC. Statistical Methods for Rates and Proportions. Third Edition. John Wiley & Sons. New York. 2003.

[24] Machin D, Campbell M, Fayers P, Pinol A. Sample Size Tables for Clinical Studies, 2nd Edition. Blackwell Science. Malden, Mass. 1997.

[25] Ryan TP. Sample Size Determination and Power. John Wiley & Sons. Hoboken, New Jersey. 2013.
