# Supplementary material for: Transcutaneous Auricular Vagus Nerve Stimulation for Postpartum Contraction Pain During Elective Cesarean Delivery: A Randomized Clinical Trial
Source: JAMA Netw Open. 2025 Aug 29;8(8):e2529127. doi: 10.1001/jamanetworkopen.2025.29127 (PMC12397895; doi:10.1001/jamanetworkopen.2025.29127)
Supplement: Supplement 2. — eTable. Binary Logistic Regression Analysis to Identify Moderate to Severe Contraction Pain at T7 eFigure 1. Apparatus Used in This Test eFigure 2. Timeline of the Experimental Design of the Two Experimental Sessions (Active-taVNS and Sham-taVNS) eFigure 3. (A) Comparison of Contraction Pain Scores Between Groups; (B) Comparison of Contraction Pain Intensity Between Groups; (C) Comparison of Incisional Pain Scores Between Groups; (D) Comparison of Incisional Pain Intensity Between Groups eFigure 4. (A)(B) Comparison of AUC of Contraction Pain in Time Periods T1-T7 Between Groups; (C)(D) Comparison of AUC of Incisional Pain in Time Periods T1-T7 Between Groups eFigure 5. Comparison of EPDS, PRAQ-R2, LSEQ and ObsQoR-11 eFigure 6. Forest Plot of the Subgroup Analysis [file jamanetwopen-e2529127-s002.pdf]

Supplementary Online Content

Xiong X, Tao M, Zhao W, et al. Transcutaneous auricular vagus nerve stimulation for postpartum contraction pain during elective cesarean delivery: a randomized clinical trial. *JAMA Netw Open.* 2025;8(8):e2529127. doi:10.1001/jamanetworkopen.2025.29127

eTable. Binary Logistic Regression Analysis to Identify Moderate to Severe Contraction Pain at T7
eFigure 1. Apparatus Used in This Test
eFigure 2. Timeline of the Experimental Design of the Two Experimental Sessions (Active-taVNS and Sham-taVNS)
eFigure 3. (A) Comparison of Contraction Pain Scores Between Groups; (B) Comparison of Contraction Pain Intensity Between Groups; (C) Comparison of Incisional Pain Scores Between Groups; (D) Comparison of Incisional Pain Intensity Between Groups
eFigure 4. (A)(B) Comparison of AUC of Contraction Pain in Time Periods T1-T7 Between Groups; (C)(D) Comparison of AUC of Incisional Pain in Time Periods T1-T7 Between Groups
eFigure 5. Comparison of EPDS, PRAQ-R2, LSEQ and ObsQoR-11
eFigure 6. Forest Plot of the Subgroup Analysis

This supplementary material has been provided by the authors to give readers additional information about their work.

eTable. Binary Logistic Regression Analysis to Identify Moderate to Severe Contraction Pain at T7

Abbreviation: OR, odds ratio; CI, confidence interval; NA, not applicable (the multivariable section in the

Variable[a]	Univariable analysis<br>Unadjusted OR (95% CI)	*P*-Value	Multivariable analysis<br>Adjusted OR (95% CI)	*P*-Value
Group	0.14 (0.05, 0.42)	＜.001	0.12 (0.04, 0.37)	＜.001
Parity	5.21 (1.85, 14.67)	0.002	4.21 (1.13, 15.71)	0.03
Dysmenorrhea	0.50 (0.20, 1.28)	0.15	1.02 (0.31, 3.37)	0.98
Breast feeding	2.89 (1.03, 8.15)	0.05	2.44 (0.77, 7.77)	0.13
Contraction pain (T1)	4.28 (0.55, 33.48)	0.17	1.65 (0.18, 15.18)	0.66
Preoperative anxiety (T0)[b]	2.12 (0.83, 5.39)	0.12	1.88 (0.65, 5.44)	0.24
Age	1.48 (0.59, 3.75)	0.41	NA	NA
ASA	1.72 (0.33, 9.05)	0.52	NA	NA
BMI	1.08 (0.29, 4.00)	0.91	NA	NA
Prenatal contraction	1.06 (0.46, 2.48)	0.89	NA	NA
Newborn weight	0.75 (0.16, 3.54)	0.72	NA	NA
Post-operative oxytocin (D3)	0.86 (0.37, 1.99)	0.72	NA	NA
Preoperative depression (T0)	0.27 (0.03, 2.09)	0.21	NA	NA
PSQI	1.39 (0.58, 3.34)	0.47	NA	NA

model only used variables with *P* value in univariable analysis <.20); ASA, American Society of Anesthesiologists physical status classification; BMI, body mass index; PSQI, Pittsburgh Sleep Quality Index; D3, the third day after operation; T0, one day before operation; T1, before the first taVNS intervention on the day of operation; T7, the third day after operation; PRAQ-R2, pregnancy anxiety questionnaire-revised-2.

[a] Specific subgroups: parity (<1 vs. ≥1), dysmenorrhea (yes vs. no), breast feeding (yes vs. no), contraction pain (T1) (<4 vs. ≥4) , age (<35 vs. ≥35), ASA score (II vs. III), BMI (<25 vs. ≥25), prenatal contraction (yes vs. no), Newborn weight (<4000 vs. ≥4000), post-operative oxytocin (D3) (yes vs. no), preoperative depression (T0) (<13 vs. ≥13), PSQI (<7 vs. ≥7).

[b] Primiparous women with PRAQ-R scores of ≥26 and parous women with PRAQ-R scores of ≥21 are considered to be suffering from anxiety.

© 2025 Xiong X et al. *JAMA Network Open.*

eFigure 1. Apparatus Used in This Test

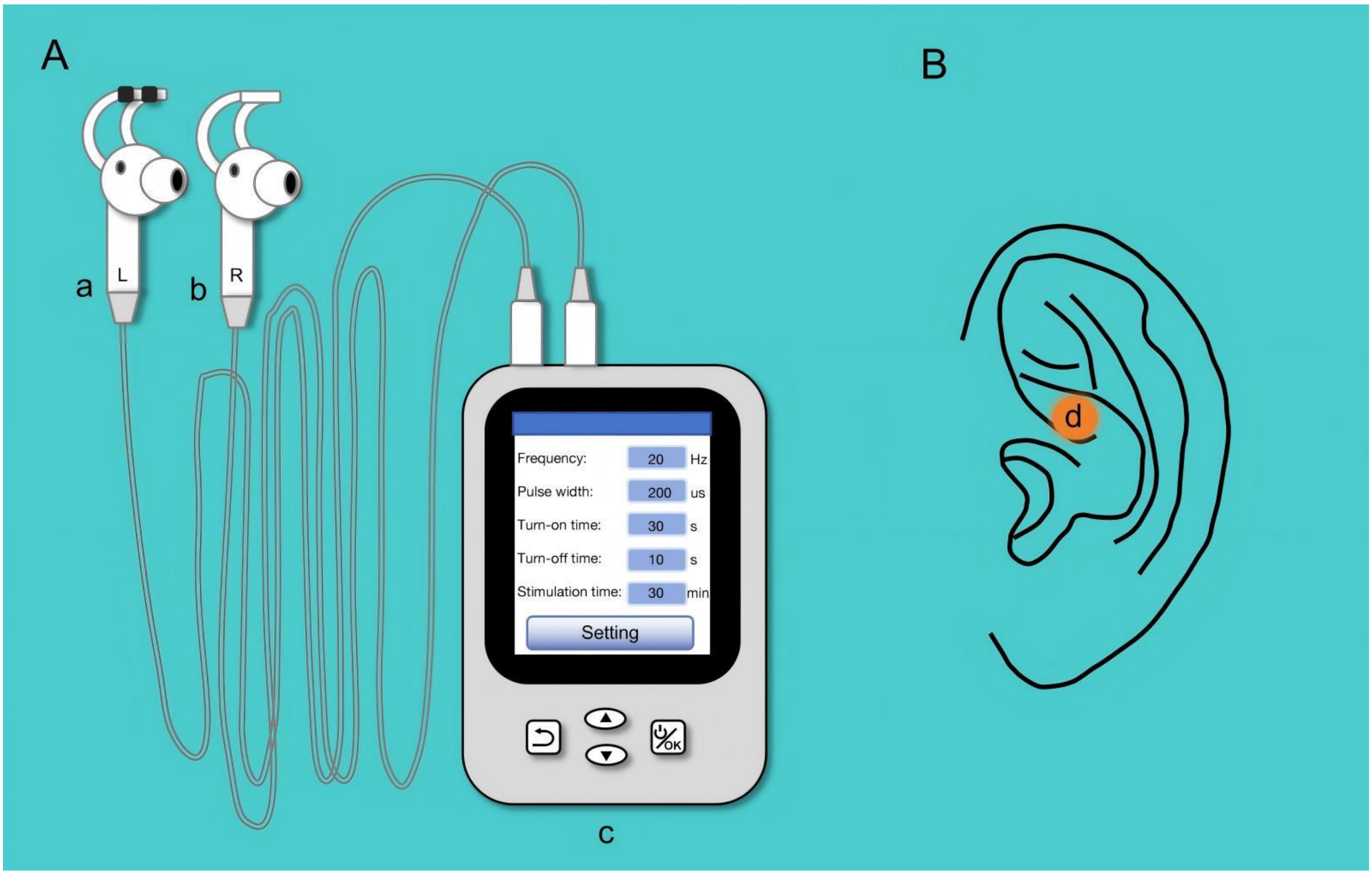

A.The apparatus used in this test. a, electrode; b, electrode earplug; c, stimulator main unit.
B.The site of stimulation. d, cymba conchae.

© 2025 Xiong X et al. *JAMA Network Open.*

eFigure 2. Timeline of the Experimental Design of the Two Experimental Sessions (Active-taVNS and Sham-taVNS)

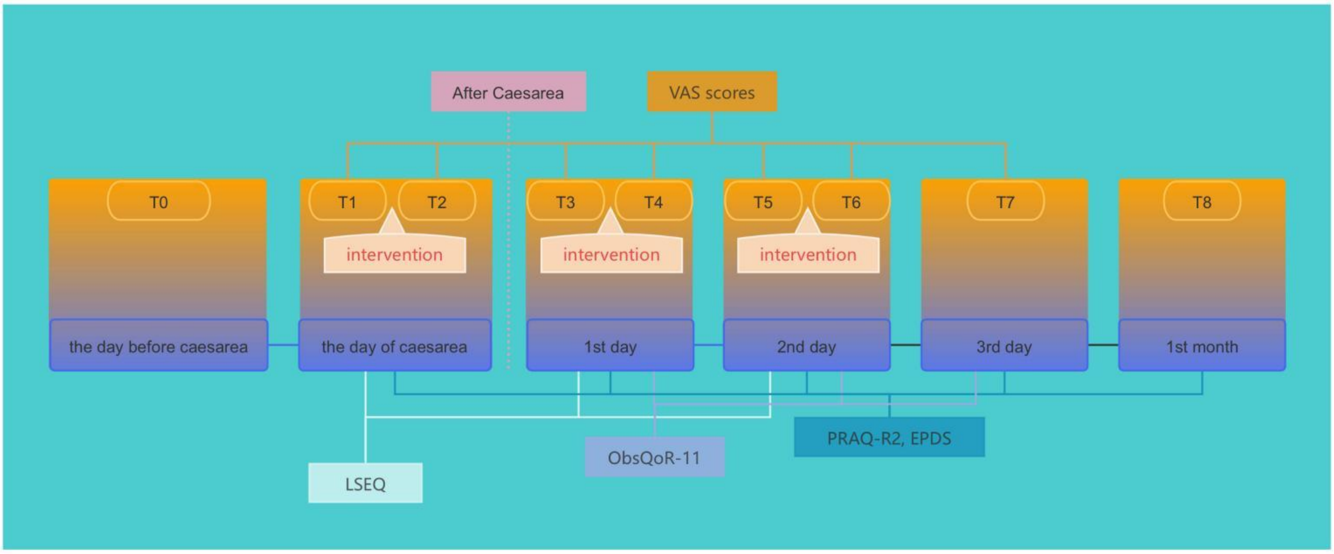

Abbreviations: taVNS, transcutaneous auricular vagus nerve stimulation; VAS, visual analogue scale; EPDS, Edinburgh postnatal depression scale; PRAQ-R2, pregnancy anxiety questionnaire-revised-2; ObsQoR-11, obstetric quality-of-recovery score; LSEQ, leeds sleep evaluation questionnaire. T0, one day before operation; T1, before the first taVNS intervention on the day of operation; T2, after the first taVNS intervention on the day of operation; T3, before the second taVNS intervention on the first day after operation; T4, after the second taVNS intervention on the first day after operation; T5, before the third taVNS intervention on the second day after operation; T6, after the third taVNS intervention on the second day after operation; T7, the third day after operation; T8, one month after operation.

© 2025 Xiong X et al. *JAMA Network Open.*

eFigure 3. (A) Comparison of Contraction Pain Scores Between Groups; (B) Comparison of Contraction Pain Intensity Between Groups; (C) Comparison of Incisional Pain Scores Between Groups; (D) Comparison of Incisional Pain Intensity Between Groups

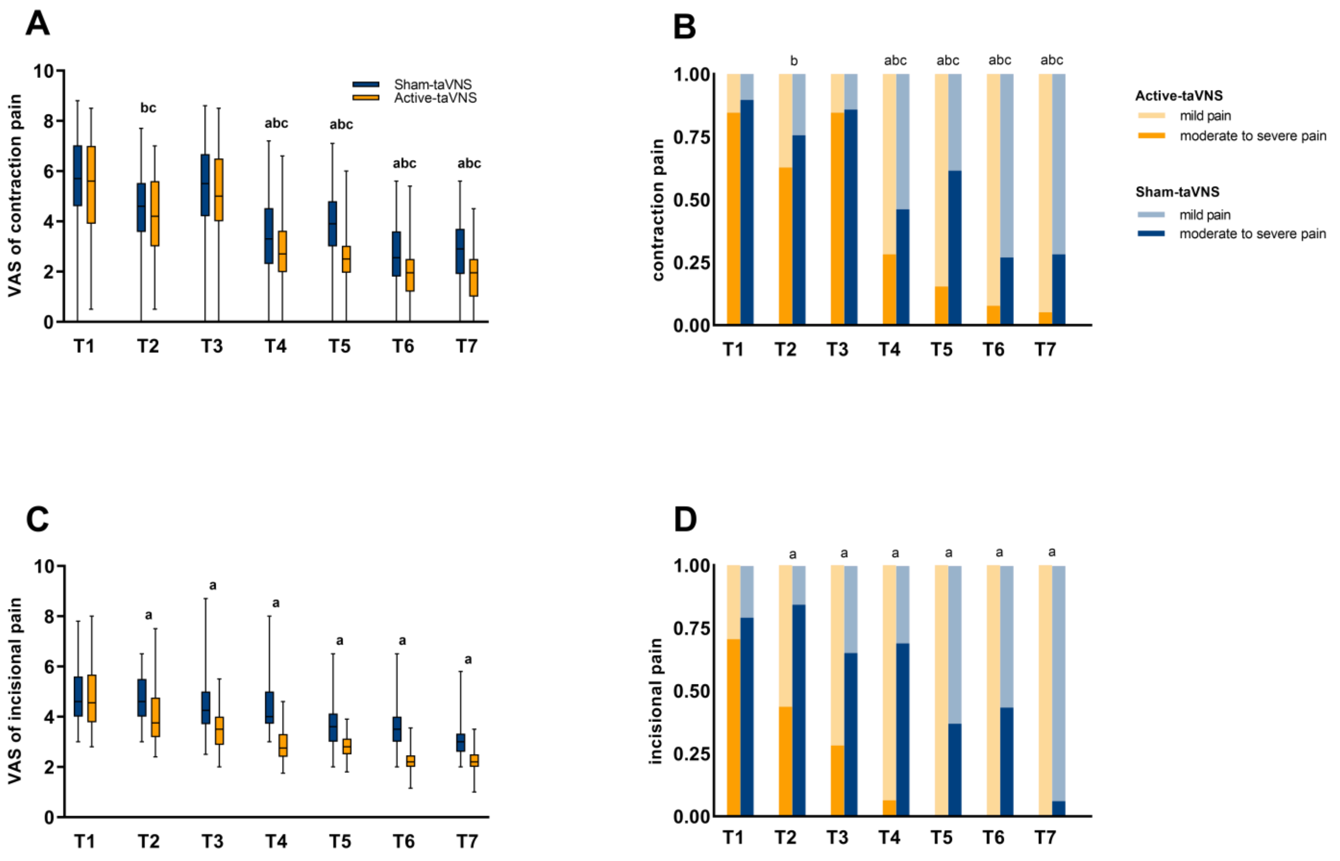

Median values shown as solid line within box of 25th and 75th percentile values. Whiskers represent maximum and minimum values.

Abbreviations: taVNS, transcutaneous auricular vagus nerve stimulation; VAS, visual analogue scale; T1, before the first taVNS intervention on the day of operation; T2, after the first taVNS intervention on the day of operation; T3, before the second taVNS intervention on the first day after operation; T4, after the second taVNS intervention on the first day after operation; T5, before the third taVNS intervention on the second day after operation; T6, after the third taVNS intervention on the second day after operation; T7, the third day after operation.

a refers to $P < .05$, when active-taVNS group vs. sham-taVNS group at each time point.

b refers to $P < .001$, when each time point vs T1 within the active-taVNS group.

c refers to $P < .001$, when each time point vs T1 within the sham-aVNS group.

© 2025 Xiong X et al. *JAMA Network Open.*

eFigure 4. (A)(B) Comparison of AUC of Contraction Pain in Time Periods T1-T7 Between Groups; (C)(D) Comparison of AUC of Incisional Pain in Time Periods T1-T7 Between Groups

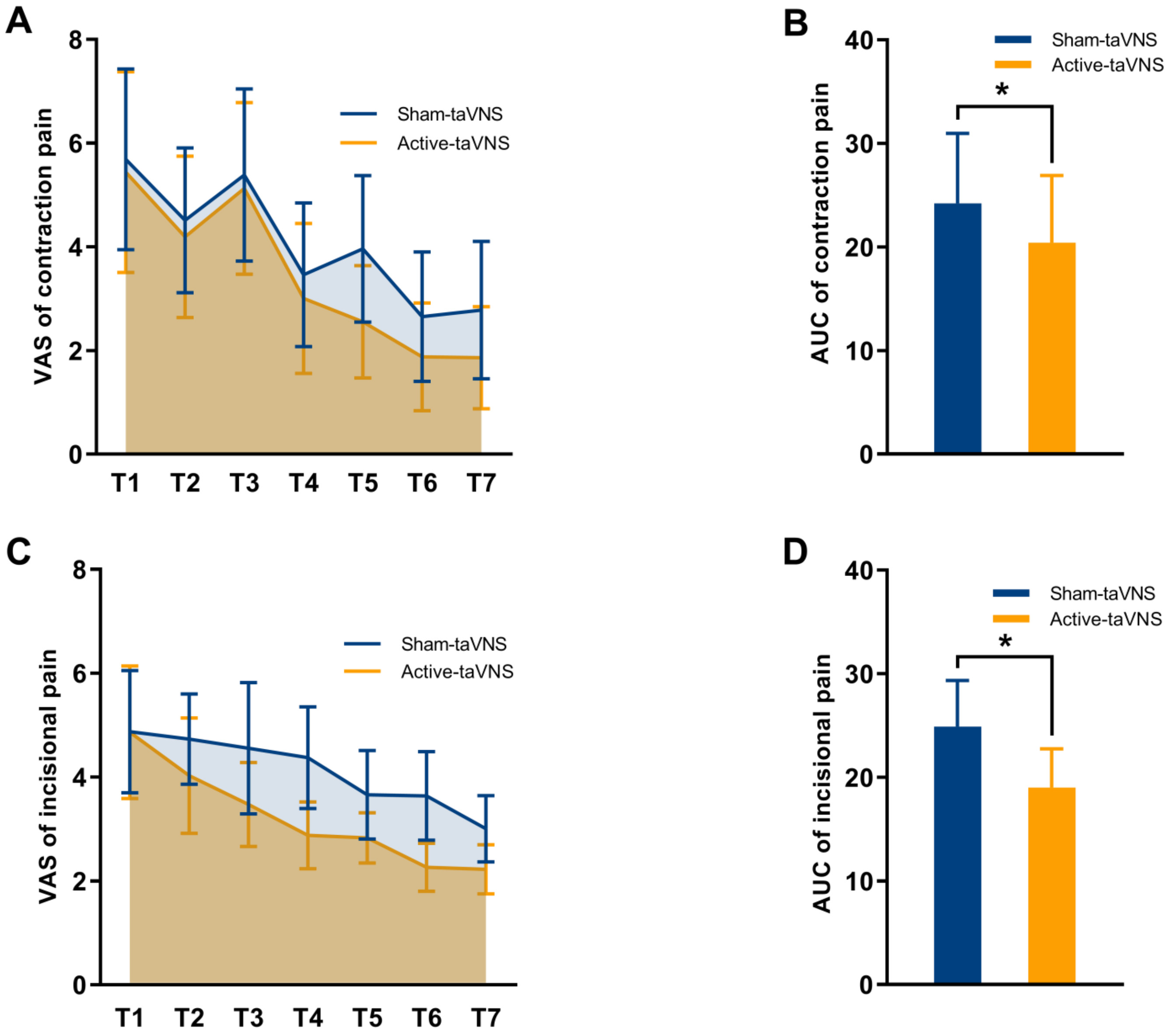

Abbreviations: taVNS, transcutaneous auricular vagus nerve stimulation; VAS, visual analogue scale; AUC, area under the curve; T1, before the first taVNS intervention on the day of operation; T2, after the first taVNS intervention on the day of operation; T3, before the second taVNS intervention on the first day after operation; T4, after the second taVNS intervention on the first day after operation; T5, before the third taVNS intervention on the second day after operation; T6, after the third taVNS intervention on the second day after operation; T7, the third day after operation.

* Refers to *P* <0.05, when active taVNS group vs. sham taVNS group.

© 2025 Xiong X et al. *JAMA Network Open.*

eFigure 5. Comparison of EPDS, PRAQ-R2, LSEQ and ObsQoR-11

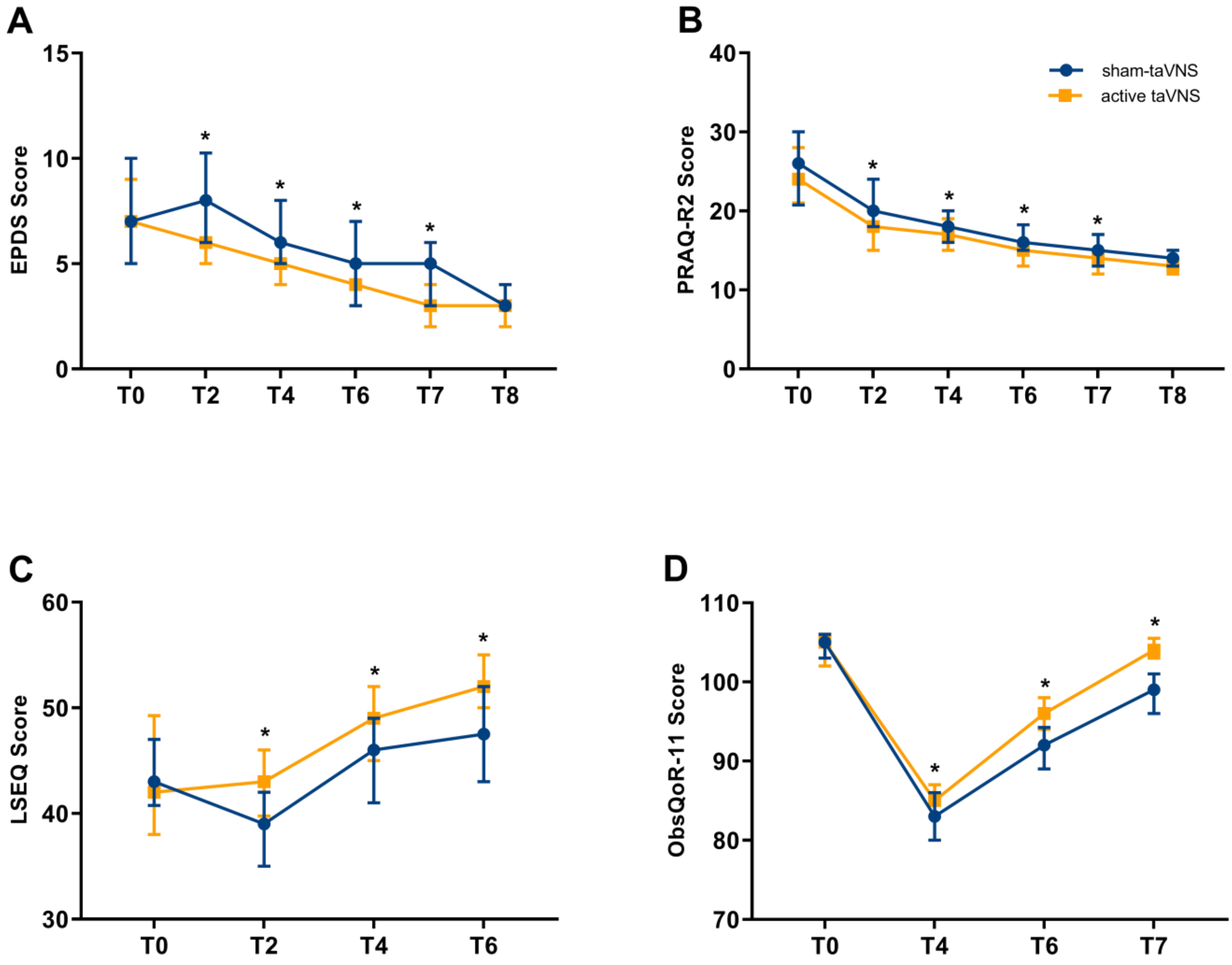

Between the Two Groups Error bars represent the median and IQR.

Abbreviation: taVNS, transcutaneous auricular vagus nerve stimulation; EPDS, Edinburgh postnatal depression scale; PRAQ-R2, pregnancy anxiety questionnaire-revised-2; LSEQ, leeds sleep evaluation questionnaire; ObsQoR-11, obstetric quality-of-recovery score. T0, one day before operation; T2, after the first taVNS intervention on the day of operation; T4, after the second taVNS intervention on the first day after operation; T6, after the third taVNS intervention on the second day after operation; T7, the third day after operation; T8, one month after operation.

* Refers to *P* <0.05, when active taVNS group vs. sham taVNS group at each time point.

© 2025 Xiong X et al. *JAMA Network Open.*

eFigure 6. Forest Plot of the Subgroup Analysis

Subgroup	Active taVNS (Events/Total)	Sham taVNS (Events/Total)	Odds ratio(95% CI)	*P* interaction
**Multipara**				0.033
Yes	3/37	18/21	0.095 (0.025-0.359)	
No	1/37	4/35	0.236 (0.025-2.220)	
**Dysmenorrhea**				0.977
Yes	1/28	6/27	0.161 (0.018-1.425)	
No	3/46	16/29	0.118 (0.032-0.442)	
**Breast feeding**				0.130
Yes	3/49	18/28	0.095 (0.026-0.352)	
No	1/25	4/28	0.280 (0.029-2.674)	
**Contraction pain (T1)**				0.656
Yes ≥ 4	4/62	21/49	0.151 (0.048-0.467)	
No < 4	0/12	1/7		
**Anxiety (T0)**				0.244
Yes	4/42	15/31	0.197 (0.059-0.651)	
No	0/32	7/25		

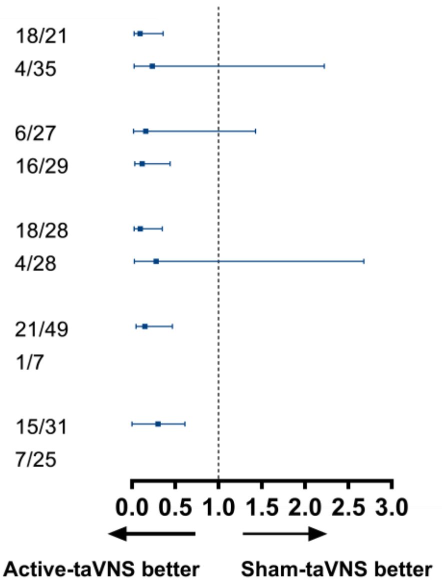

Abbreviation: taVNS, transcutaneous auricular vagus nerve stimulation; T0, one day before operation; T1, before the first taVNS intervention on the day of operation.

Forest plot assessing the effect of combined supplement versus placebo in predefined subgroups. Logistic models were applied for assessment of treatment-by-covariate interactions. Treatment-by-covariate interactions were assessed separately for each subgroup factor, including Parity, dysmenorrhea, breast feeding, contraction pain (T1) and anxiety (T0). The effect of the intervention method (odds ratio [95% CI]) is presented separately in each subgroup. The interaction term is a test of whether the effect of the experimental intervention is statistically different in significance between subgroups.

© 2025 Xiong X et al. *JAMA Network Open.*
