# Supplementary material for: Transcutaneous Auricular Vagus Nerve Stimulation for Postpartum Contraction Pain During Elective Cesarean Delivery: A Randomized Clinical Trial
Source: JAMA Netw Open. 2025 Aug 29;8(8):e2529127. doi: 10.1001/jamanetworkopen.2025.29127 (PMC12397895; doi:10.1001/jamanetworkopen.2025.29127)
Supplement: Supplement 3. — Data Sharing Statement [file jamanetwopen-e2529127-s003.pdf]

Data Sharing Statement

Xiong. Transcutaneous Auricular Vagus Nerve Stimulation for Postpartum Contraction Pain During Elective Cesarean Delivery. *JAMA Netw Open*. Published August 29, 2025. doi:10.1001/jamanetworkopen.2025.29127

Data
Additional Information: Chinese Clinical Trial Register Identifier: ChiCTR2400082716
Data available: No
